# The Effect of Nutrition Impact Symptoms on Nutrition Status After Completion of Curative-Intent Treatment for Gastric, Oesophageal, and Pancreatic Cancer: A Systematic Review

**DOI:** 10.3390/nu17162691

**Published:** 2025-08-20

**Authors:** Emma McShane, Lauren Hanna, Carmel Zoanetti, Lisa Murnane, Brenton Baguley, Kate Furness

**Affiliations:** 1 Department of Sport, Exercise and Nutrition Sciences, School of Allied Health, Human Services and Sport, La Trobe University, Bundoora, VIC 3086, Australia; e.mcshane@alfred.org.au; 2 Nutrition & Dietetics Department, Alfred Health, Melbourne, VIC 3004, Australia; c.zoanetti@alfred.org.au (C.Z.); l.murnane@alfred.org.au (L.M.); 3 Department of Nutrition, Dietetics and Food, Faculty of Medicine Nursing and Health Sciences, Monash University, Notting Hill, VIC 3168, Australia; lauren.hanna@monash.edu; 4 Department of Surgery, Central Clinical School, Monash University, Alfred Health, Melbourne, VIC 3004, Australia; 5 Institute for Physical Activity and Nutrition, Deakin University, Melbourne, VIC 3125, Australia; b.baguley@deakin.edu.au; 6 Allied Health Research, Peter MacCallum Cancer Centre, Melbourne, VIC 3051, Australia

**Keywords:** nutrition impact symptoms, nutrition outcomes, upper gastrointestinal cancers, post-treatment, pancreatic cancer, gastric cancer, oesophageal cancer

## Abstract

**Background/Objectives**: Gastric, oesophageal, and pancreatic cancers are common worldwide, with low but improving survival rates due to advances in curative treatments. However, these treatments often cause long-term nutrition impact symptoms that are frequently overlooked, leading to malnutrition and poorer health outcomes. This review explored the types of nutrition impact symptoms following curative-intent treatment for upper gastrointestinal (UGI) cancers and assessed their impact on nutrition status. **Methods:** A systematic search of four electronic databases identified studies involving adults treated with curative intent for UGI cancers. Included studies reported both nutrition impact symptoms and nutrition outcomes using validated tools. Study quality was assessed, and results were synthesised narratively. **Results:** Eleven studies (n = 953 participants), predominantly from the Asia–Pacific region, met the inclusion criteria. Participants were mostly male (68%), with surgery as the primary treatment (91%). Most studies (n = 10) used validated assessment tools to identify nutrition impact symptoms. Reflux was the most commonly reported symptom (n = eight studies), followed by abdominal pain and diarrhoea. Nutrition status was assessed using various validated tools: Patient-Generated Subjective Global Assessment (n = six studies, 55%), Mini Nutritional Assessment (n = two studies, 18%), and Global Leadership Initiative on Malnutrition as well as Subjective Global Assessment and Prognostic Nutritional Index (both n = one study, 9%). Malnutrition prevalence was up to 87% one year post-treatment. **Conclusions:** Nutrition impact symptoms are common and persistent after curative-intent treatment for UGI cancers. Future research should incorporate validated assessment tools and extend follow-up beyond 12 months. Integrating long-term, tailored dietetic support to identify and manage symptoms post-treatment is vital to improve outcomes for patients.

## 1. Introduction

Cancers of the upper gastrointestinal (UGI) tract, including gastric, oesophageal, and pancreatic cancers, account for 26% of the global cancer incidence burden [1,2], with close to two million people diagnosed in 2022 [1]. Globally, the published five-year survival rates range from 21 to 33% for gastric, 15–23% for oesophageal, and 8–15% for pancreatic cancers [3]. Survival has improved across all stages of UGI cancers, with treatments advancing in recent years [4,5]. Curative treatment within these cancer types may involve neoadjuvant chemoradiotherapy and surgeries such as gastrectomy, oesophagectomy, or pancreaticoduodenectomy, followed by adjuvant chemotherapy with or without radiotherapy, immunotherapy, or other targeted therapies. Improvements in these modalities have led to a larger population of survivors [6,7,8]. Although surgery is often successful in removing a tumour, an individual’s quality of life (QoL) may be negatively impacted by symptoms that occur during and long after treatment completion [4,6].

Nutrition impact symptoms affect an individual’s ability to eat and drink, and are the result of a combination of the cancer itself (tumour location, stage of disease) and its treatment [9,10]. Common symptoms include anorexia, dysphagia, xerostomia (dry mouth), nausea, and vomiting [10]. Irreversible gastrointestinal anatomical changes post-surgical resection can cause lifelong symptoms due to the loss of digestive or absorptive mechanisms and can affect the ability to tolerate food and fluid [11]. Pancreatic enzyme insufficiency (PEI) is common in patients with pancreatic cancer. In those who have undergone surgery to remove part of the pancreas, known as pancreaticoduodenectomy or Whipple’s procedure, the prevalence of PEI is 74% [12]. If PEI is left untreated without effective management, it can lead to detrimental symptoms of bloating, steatorrhea (fatty stools), weight loss, and deficiencies in fat-soluble vitamins [6,11]. Similarly, partial or total removal of the stomach and/or oesophagus impacts the volume of food and fluid able to be consumed and can lead to symptoms of dumping syndrome, nausea, early satiety, and PEI due to maldigestion and malabsorption [13,14,15]. Treatments such as chemotherapy, radiotherapy, and immunotherapy often cause symptoms such as nausea, vomiting, and diarrhoea [16,17]. Untreated, these symptoms can result in cancer-related malnutrition, sarcopenia, cancer cachexia, and reduced QoL [11].

Due to the location of these tumours and the long-term nutrition impact symptoms related to their treatment, gastric, oesophageal, and pancreatic cancers are amongst the highest causes of disease-associated malnutrition, sarcopenia, and cancer cachexia [15,18]. Malnutrition is a condition resulting from the inadequate consumption or absorption of nutrients and/or as the result of inflammation or chronic disease, with accompanying weight loss, low body mass index (BMI), and/or loss of muscle mass [19]. Depending on the method of assessment, the prevalence of malnutrition is as high as 40–80% in the general cancer population [18,20]. Recent literature suggests global rates of malnutrition were highest amongst UGI cancers, with a prevalence of around 70–78% amongst gastric, oesophageal, and pancreatic cancers [21,22,23]. Malnutrition can occur simultaneously with sarcopenia. The prevalence of sarcopenia, defined as the loss of muscle strength with the loss of muscle mass [24], is also high, with rates estimated to be around 32–70% for gastric, oesophageal, and pancreatic cancers [25]. If malnutrition and sarcopenia are left untreated, this can result in cancer cachexia [26]. Cancer cachexia is a multisystem metabolic disease demonstrating the progressive loss of muscle mass (with or without the loss of fat mass), insulin resistance, and systemic inflammation [19,24,27]. These interconnected conditions, if left unmanaged, lead to increases in treatment-related toxicities, dose-limiting toxicity during chemotherapy, and postoperative complications, as well as a significant reduction in QoL and increases in cancer-related death [19].

Despite the high prevalence of post-treatment nutrition impact symptoms and associated malnutrition, sarcopenia, and cancer cachexia, there is limited standardised practice to monitor and manage these symptoms over the long term [28]. Identifying and managing nutrition impact symptoms post-treatment is important to help reduce the risk of patients developing malnutrition, sarcopenia, and cancer cachexia. Access to specialised dietitian involvement within the long-term post-cancer treatment group is needed, with dietetic services currently usually ceasing post-discharge from hospital or oncological services [4,5]. The aim of this review is to characterise the nutrition impact symptoms occurring across UGI cancers and explore the relationship between nutrition impact symptoms and nutrition status after curative treatment for gastric, oesophageal, and pancreatic cancers.

## 2. Materials and Methods

This systematic review was reported according to the Preferred Reporting Items for Systematic reviews and Meta-Analysis (PRISMA) guidelines [29]. This review was prospectively registered with the International Prospective Register of Systematic Reviews (PROSPERO) on 24 February 2025 (CRD420250649071).

### 2.1. Data Sources and Search Strategy

A systematic search of the Ovid MEDLINE, Embase via Ovid, Scopus, and Cochrane Library databases was conducted on 11 February 2025. A combination of keywords and subject headings, such as ‘nutrition impact symptoms’, ‘nutrition status’, ‘cancer’, and ‘treatment’ were used for each database. Truncations and Boolean Operators were applied to relevant keywords and subject headings for each database. Details of search terms for each database are available in the Supporting Information, Supplementary File S1. The search was run from January 2020 until present to include all relevant studies published within this time frame. This date range was chosen for the inclusion of studies, given the rapid emergence of contemporaneous treatment modalities such as surgical techniques and chemotherapy regimens to ensure that the review reflects the most clinically relevant up-to-date evidence.

### 2.2. Study Selection Criteria

**Figure 1:**
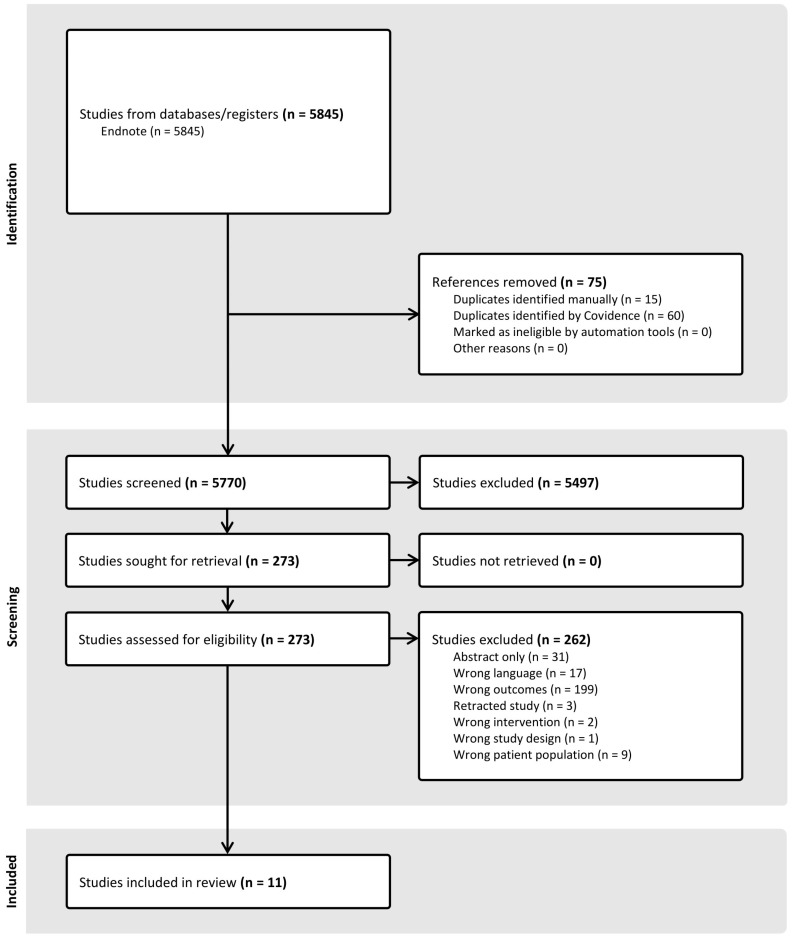
PRISMA Flow Diagram.

Full-text studies reporting on the effect of long-term nutrition impact symptoms (>30 days from treatment completion) (Intervention) on nutrition status (Outcomes) in adults aged over 18 years undergoing curative-intent treatment for gastric, oesophageal, and pancreatic cancer (Population) were eligible for inclusion. Analysis of the effect of nutrition impact symptoms (with or without a validated tool) on nutrition status (using a validated tool) at the baseline was considered the primary outcome. Characterisation of long-term nutrition impact symptoms in curative-intent gastric, oesophageal, and pancreatic cancers was the secondary outcome. Studies not published in the English language, conference abstracts, and letters to the editor were excluded. A systematic search, database retrieval, and de-duplication were conducted by the first researcher (E.M.). Studies retrieved were imported to EndNote (Version 21) [30], where a process of de-duplication occurred using the Bramer method of de-duplication [30,31]. De-duplicated studies were then imported to Covidence^®^, where additional duplications were removed [32]. Article titles and abstracts were independently screened by the authorship team using Covidence^®^ systematic review software [32]. Full-text articles were then independently reviewed for inclusion against the eligibility criteria. The reasons for exclusion were documented and are presented in Figure 1. Reference lists of all studies meeting the inclusion criteria were searched by hand. At each stage, consensus was achieved through discussion prior to progression of screening.

### 2.3. Data Extraction

A template was created in Excel for data extraction. A researcher (E.M.) extracted data relating to study characteristics: author, year of publication, study design, country of origin, setting, patient demographics (number of participants, cancer type and stage, age, and gender), nutrition impact symptoms (type, assessment frequency, and validation tool use (yes/no, type)), and nutrition status (type, frequency of assessment, and validated tool use). A second researcher (B.B.) independently extracted primary outcome data related to study design, nutrition impact symptoms, and nutrition status for four studies. Discrepancies in data extraction were addressed by a repeat review of relevant studies and discrepancies were resolved through a consensus discussion between the two reviewers to ensure accuracy.

### 2.4. Result Synthesis

This review presents a narrative synthesis of the results reported by the selected studies, with the use of the Synthesis Without Meta-analysis (SWiM) in systematic reviews: reporting guidelines are in Supplementary File S2 [33]. The narratively synthesised results present the effect of nutrition impact symptoms on nutrition status assessed with a validated tool. Where possible, we grouped treatment arms to report on a whole number for nutrition impact symptoms and nutrition status [34,35,36,37]. For studies where this could not be performed, we reported individual treatment arms for nutrition impact symptoms and nutrition status.

### 2.5. Quality Assessment

All included studies were assessed for quality and risk of bias. One researcher (E.M.) determined the risk of bias for all of the studies, whilst the other members in the research team completed three studies, with one researcher completing two studies (K.F., B.B., L.H., and C.Z.) using the Academy of Nutrition and Dietetics Quality Checklist for Primary Research [38]. The criteria for the classification of negative were established if the majority of the answers to validity questions (six or more) were “no”, whereas a positive rating occurred when most of the answers to the validity questions (at least six or more) were “yes”. A neutral rating was established when the answers to questions 2, 3, 6, and 7 did not suggest an overly strong study as guided by the checklist [38]. Assessments were undertaken independently, and a consensus was formed through author group discussion to resolve conflicts.

## 3. Results

### 3.1. Study Selection

A total of 7926 studies were initially identified through searches across four databases. After removing 2141 duplicate records, 5785 studies remained for title and abstract screening. Of these, 273 studies proceeded to full-text review. During this stage, 262 studies were excluded for the following reasons: wrong outcomes (n = 199), abstract only (n = 31), wrong language (n = 17), wrong patient population (n = 9), retracted study (n = 3), wrong intervention (n = 2), and wrong study design (n = 1). Eleven studies met the inclusion criteria and were included in the final review.

### 3.2. Study Characteristics

Characteristics of the studies included are presented in Table 1.

A total of 953 participants were recruited in the included studies between 2012 and 2023, with sample sizes ranging from n = 21 [37] to n = 409 [39]. Studies were conducted in China (n = five studies) [35,39,43,44,45], Taiwan (n = three studies) [40,41,42], Norway [37], Romania [34], and Japan [36] (all n = one study). Study designs varied, with five retrospective cohort studies [35,36,39,43,44], three cross-sectional studies [34,37,42], one non-randomised controlled trial [40], one case series [45], and one prospective cohort study [41]. Seven studies included participants with gastric cancer [34,35,36,37,39,42,43], two studies reported gastric and esophagogastric cancer together [44,45], one study involved pancreatic cancer [41], and one study included oesophageal cancer [40]. Ten out of the eleven studies examined variations in surgery techniques/procedures [34,35,36,37,39,41,42,43,44,45], whilst the other study involved patients undergoing chemoradiotherapy [40].

Participants ranged in age from 25 to 89 years, and the majority were male (n = 652, 68%). All included studies collected data on participant characteristics, followed by outcome assessments at varying timepoints, with the longest follow-up period extending to 12 months post-baseline data collection.

### 3.3. Quality Assessment

**Figure 2:**
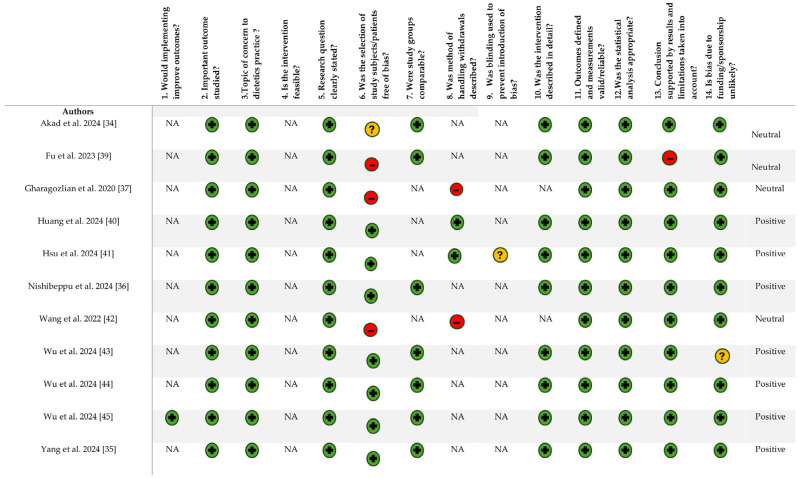
Quality and Risk of Bias using Academy of Nutrition and Dietetics Quality Checklist for Primary Research [34,35,36,37,39,40,41,42,43,44,45]. ‘+’ indicates yes for the question, ‘−‘ indicates no and ‘?’ indicates unclear.

A summary of the quality assessments for the 11 included studies is presented in Figure 2. Four studies were assessed as neutral [34,37,39,42], while the remaining studies received positive ratings [35,36,40,41,43,44,45]. Across all studies, the relevance of the research topic to patients, participants, or the population, as well as the focus of the interventions with common concerns in practice, were consistently identified as strengths. Additional strengths included clearly defined research questions, valid and reliable outcome measures, and the use of appropriate statistical analysis methods. Limitations included the lack of bias and insufficient detail in describing the handling of withdrawals. These limitations may reduce the certainty of the findings given the high risk of bias. The use of blinding and the feasibility of the studies were generally not applicable.

### 3.4. Nutrition Impact Symptoms

A summary of the nutrition impact symptoms described in each study is presented in Table 2.

### 3.5. Reflux

Reflux was the most prevalent nutrition impact symptom described, with seven out of eleven studies investigating reflux [35,36,37,42,43,44,45]. Wu et al. [43] reported reflux scores of 3.0–4.1 (range of 1 to 7, where higher scores indicate higher severity) using the Postgastrectomy Syndrome Assessment Scale-45 (PGSAS-45) tool [46] among participants undergoing two different surgical interventions for laparoscopic proximal gastrectomy. A statistically significant increase in reflux was seen between these two surgical groups (*p* = 0.006) [43]. This study also used the Los Angeles Classification Scale [47] to assess reflux oesophagitis, and reported two cases in the side overlap anastomosis (SOA) group and nine cases in the double-tract anastomosis (DTA) group after laparoscopic proximal gastrectomy, displaying a significant increase within the DTA group (*p* = 0.029).

Nishibeppu et al. [36] used the Postgastrectomy Syndrome Assessment Scale-37 (PGSAS-37) [48] to compare normal/moderate malnutrition with patients who were severely malnourished as measured by the Global Leadership Initiative on Malnutrition (GLIM) [49]. The results revealed that mean scores were the same between the two groups [36]. Yang et al. [35] compared the presence of reflux oesophagitis between two surgical techniques for proximal subtotal gastrectomy without using an assessment tool and found that the prevalence varied (from 0 to 5.6%) depending on the surgical technique used (where a higher percentage was seen in the laparoscopic surgical group compared to the Da Vinci robotic surgical group). Wu et al. [44] used three tools—the Gastroesophageal Reflux Disease scale (GERD scale) [50], PGSAS-45, and the Los Angeles Classification scale—to assess reflux, finding that reflux was still present six and twelve months postoperatively regardless of the surgical technique used for laparoscopic proximal gastrectomy [44]. Wang et al. [42] assessed reflux using the Gastric Cancer subscale of the Functional Assessment of Cancer Therapy-Gastric (FACT-Ga) [51], and reported a low reflux score following surgery (total or subtotal gastrectomy) [42]. Gharagozlian et al. [37] used the Gastrointestinal Symptom Rating Scale (GSRS) [52] to evaluate reflux symptoms amongst other nutrition impact symptoms post-surgery (total or subtotal gastrectomy) [37]. Reflux scores were higher in malnourished patients compared to well-nourished patients; however, no statistically significant difference was observed [37]. Another study by Wu et al. [45] reported that reflux symptoms remained stable at six and twelve months post-surgery (laparoscopic proximal gastrectomy) when using the GERD scale, and no signs of oesophagitis at six months were found when using the Los Angeles Classification [45].

### 3.6. Abdominal Pain

Abdominal pain was identified as a nutrition impact symptom in six out of the eleven included studies [36,37,39,42,43,44]. Among these, two studies reported statistically significant differences in abdominal pain between two groups [37,39]. In the study by Gharagozlian et al. [37], participants classified as malnourished using the Subjective Global Assessment (SGA) [53] reported significantly higher abdominal pain scores compared to well-nourished individuals (*p* = 0.042). Similarly, Fu et al. [39] observed a statistically significant increase in abdominal pain between the robotic-assisted (RG) and laparoscopic-assisted (LG) surgical groups for total gastrectomy at three, six, and twelve months postoperatively (*p* = 0.003 at three months, *p* = 0.015 at six months, and *p* = 0.016 at twelve months). Fu et al. [39] also investigated the level of abdominal pain based on Patient-Generated Subjective Global Assessment (PG-SGA) [54] scores and found that participants with higher PG-SGA scores (higher risk of malnutrition) experienced greater severity of abdominal pain [39]. Two studies used the PGSAS-45 scale to assess abdominal pain [43,44]. Abdominal pain persisted for up to 12 months following treatment [43,44]. Nishibeppu et al. [36] utilised PGSAS-37 and found that abdominal pain peaked one month postoperatively (distal or total gastrectomy), regardless of nutrition status, and, though reduced, remained present at 12 months with similar levels across nutrition status groups. In the study by Wang et al. [42], abdominal discomfort was captured using the Gastric Cancer subscale of the FACT-Ga, where the item “having stomach problems that worry me” received a mean score of 1.05 out of 4 (0 = not at all, 4 = very much) post-surgery (total or subtotal gastrectomy).

### 3.7. Diarrhoea

Diarrhoea or loose stools were identified as a nutrition impact symptom in six of the eleven studies [36,37,39,42,43,44], with five different assessment tools used across these studies [47,49,52,53,55]. Two studies [43,44] employed the PGSAS-45 tool, where higher scores on the diarrhoea and loose stool subscales (seven-point Likert scale) indicate more severe symptoms. In the study by Wu et al. [43], mean scores for diarrhoea and loose stools were the same between surgical treatment groups for laparoscopic proximal gastrectomy (1.3 and 1, respectively). In a separate study by Wu et al. [44], diarrhoea was present at 12 months post-treatment in both surgical technique groups after laparoscopic proximal gastrectomy. Wang et al. [42] reported low levels of diarrhoea postoperatively using the FACT-Ga subscale. Nishibeppu et al. [36] showed that patients who were severely malnourished had a significant increase in diarrhoea compared to those in the normal/moderate malnutrition group (*p* = 0.004) using PGSAS-37. Gharagozlian et al. [37] reported similar findings, where malnourished participants reported higher levels of diarrhoea than those who were well-nourished. Similarly, when comparing diarrhoea symptom scores with nutrition status, Fu et al. [39] demonstrated that participants with a worse nutrition status had higher levels of diarrhoea. Fu et al. [39], using an integration of two tools—European Organization for Research and Treatment of Cancer (EORTC) QLQ—C30 questionnaire and EORTC QLQ—STO22 questionnaire [55,56]—observed a statistically significant increase in diarrhoea at three months when comparing RG and LG surgical techniques for total gastrectomy (*p* = 0.014) [39].

### 3.8. Constipation

Constipation was identified as a nutrition impact symptom in four studies [36,37,43,44]. In Wu et al. [43], both surgical groups reported identical mean scores for constipation measured by PGSAS-45 after laparoscopic proximal gastrectomy. Similarly, in a separate study by Wu et al. [44], also using the PGSAS-45 tool, the mean constipation scores were low for both surgical techniques after laparoscopic proximal gastrectomy. Nishibeppu et al. [36] found that constipation scores using PGSAS-37 remained stable in the normal/moderate malnutrition group between one month and one year postoperatively. In contrast, the severely malnourished group showed a slight reduction in constipation symptoms over time [36]. In the study by Gharagozlian et al. [37], those who were malnourished experienced higher levels of constipation than those who were well-nourished, using the GSRS syndrome scale [52].

### 3.9. Dysphagia

Dysphagia was identified as a nutrition impact symptom in four studies [34,39,40,42]. Huang et al. [40] assessed dysphagia using the Functional Oral Intake Scale (FOIS) tool [57], before and after concurrent chemoradiotherapy (CCRT). Although slight improvements in scores were observed post-CCRT, the average score remained 5 on the FOIS, indicating that oral intake still required special preparation and was not entirely unrestricted (0 = tube feeding, 7 = full oral intake without restrictions) [40]. Akad et al. [34] reported that 17.6% of patients experienced altered eating habits due to symptomatic dysphagia (score of 2 for observer-reported dysphagia) postoperatively, regardless of the surgery type (total and subtotal gastrectomy). Wang et al. [42] found that dysphagia was present at a low level using the Gastric Cancer Subscale of the FACT-Ga [51]. Fu et al. [39] demonstrated that malnutrition severity correlated with higher dysphagia scores as measured by a combination of two tools: the EORTC QLQ—C30 questionnaire and EORTC QLQ—STO22 questionnaire.

### 3.10. Fatigue

Fatigue was reported as a nutrition impact symptom in three studies [39,41,42], each utilising a different assessment tool [52,55,58]. Hsu et al. [41] employed the Fatigue Symptom Inventory [59], where higher scores indicate greater fatigue. Fatigue levels increased at three months postoperatively compared to before surgery (distal pancreatectomy and splenectomy, total pancreatectomy or bypass operation) but subsequently decreased at six and twelve months [41]. Fu et al. [39] also observed increased fatigue at all postoperative timepoints in both surgical groups (RG and LG surgical techniques for total gastrectomy) compared to preoperatively, using the integration of two tools: the EORTC QLQ—C30 questionnaire and EORTC QLQ—STO22 questionnaire. Fu et al. [39] also showed that fatigue scores worsened with higher scores of PG-SGA (worsening nutrition status). Wang et al. [42] reported a relatively low level of fatigue postoperatively using the FACT-Ga.

### 3.11. Dumping Syndrome

Three studies evaluated the presence and severity of dumping syndrome following treatment [36,43,44] using two different tools: PGSAS-45 and PGSAS-37. Both studies that utilised PGSAS-45 [43,44] reported that although no difference was observed between surgical technique groups after proximal laparoscopic surgery, dumping syndrome was present post-treatment. Nishibeppu et al. [36] found that scores were higher among the severely malnourished group at both one month and twelve months post-distal or total gastrectomy, compared to those classified as normal or moderately malnourished using the PGSAS-37.

### 3.12. Increased Flatus

Increased flatus was reported as a nutrition impact symptom in three studies [42,43,44]. Using the PGSAS-45 tool, Wu et al. [43] found that flatus significantly increased between surgical techniques (side overlap anastomosis (SOA) vs. double-tract anastomosis (DTA)) after proximal laparoscopic gastrectomy (*p* = 0.036). In another study by Wu et al. [44], also using PGSAS-45, flatulence ranged from 1.3 to 3.0 between surgical techniques after proximal laparoscopic gastrectomy. Wang et al. [42] assessed flatus using FACT-Ga [51], where “being bothered by gas” had the highest mean score postoperatively among all symptoms measured within this study.

### 3.13. Indigestion

Indigestion was reported as a nutrition impact symptom in three studies [36,43,44]. Wu et al. [43] showed that mean indigestion scores ranged from 2.3 to 2.5 after proximal laparoscopic gastrectomy (range of 1 to 7; higher scores indicate worse symptoms), with indigestion representing one of the highest-rated nutrition impact symptoms in the study based on PGSAS-45. Similarly, in another study by Wu et al. [44] comparing two surgical techniques for gastrectomy, there was no difference in indigestion scores. Gharagozlian et al. [37] reported higher indigestion scores among malnourished participants compared to those who were well-nourished (3.5 vs. 2.9 out of 7) using the GSRS.

### 3.14. Appetite

Two studies [35,44] assessed appetite following treatment using different validated tools: the Simplified Nutritional Assessment Questionnaire (SNAQ) score [58], where higher scores indicate better appetite [34], and the Gastric Cancer Subscale of the FACT-Ga, which rates symptoms on a four-point scale (0 = not at all, 4 = very much) [42]. Akad et al. [34] reported that approximately 6% of participants (n = 3) had medium to low appetite postoperatively based on the SNAQ score. Wang et al. [42] found minimal appetite loss among participants using the FACT-Ga.

### 3.15. Other Nutrition Impact Symptoms

Some nutrition impact symptoms were investigated in only one study within this review. Gharagozlian et al. [37] investigated bodily pain using the Short Form 36 Health Survey Questionnaire (SF-36) [60] and found that malnourished patients experienced significantly more pain postoperatively, as indicated by lower SF-36 pain scores (*p* = 0.015). Fu et al. [39] explored both sour regurgitation and belching as nutrition impact symptoms, using both the EORTC QLQ—C30 questionnaire and EORTC QLQ—STO22 questionnaire, as well differences in them between surgical techniques and malnutrition scores (using PG-SGA). Sour regurgitation increased at three months post-surgery in both surgical technique groups for a total gastrectomy, compared to preoperatively, but then in both surgical groups sour regurgitation decreased at six and twelve months [39]. When comparing sour regurgitation with PG-SGA scores, levels of sour regurgitation overall were worse when PG-SGA scores were greater than nine (indicating malnutrition) [39]. Fu et al. [39] reported that there was a significant increase in belching between the two surgical techniques at both three and six months post-surgery, and although it reduced at twelve months compared to three and six months, the levels of belching in both groups were increased compared to the preoperative data. When comparing the severity of belching based on nutrition status scores (using PG-SGA scores), the higher the malnutrition severity the greater the severity of belching. Nishibeppu et al. [36] compared glycaemic levels between participants with normal/moderate malnutrition and severe malnutrition (as diagnosed by the GLIM criteria) one year after surgery and reported that participants with severe malnutrition experienced less time in the normal glycaemic range than those who were well-nourished or moderately malnourished (normal/moderate: 79.6% vs. severe: 75.1%).

### 3.16. Nutrition Outcomes

A summary of nutrition outcomes is presented in Table 3.

### 3.17. Malnutrition Risk

Six out of the eleven studies assessed malnutrition risk at follow-up timepoints [34,35,39,43,44,45]. Five studies employed the Nutrition Risk Screening 2002 (NRS-2002) tool [61] to determine malnutrition risk [34,35,39,43,45]. Wu et al. [43] reported a reduction in malnutrition risk from three to twelve months postoperatively in both surgical groups. Yang et al. [35] reported that all participants scored a low risk of malnutrition in both groups postoperatively. Similarly, Akad et al. [34] found that most patients had a low nutrition risk using NRS-2002 [34]. Fu et al. [39] reported that 34.9–40.9% of participants were at risk of developing malnutrition across treatment group (NRS 2002 score ≥ 3). Wu et al. [45] observed no statistically significant change in nutrition risk over time, with scores remaining stable (mean score = 2). One study used the Malnutrition Universal Screening Tool (MUST) [62] to assess nutrition risk (1 = low malnutrition risk, 2 = moderate malnutrition risk, and 3 = high malnutrition risk) at six and twelve months post-treatment [44]. No changes were observed over time, with both surgical groups maintaining a moderate MUST score at all follow-up timepoints [44].

### 3.18. Nutrition Status

Six out of eleven studies assessed malnutrition using the Patient-Generated Subjective Global Assessment (PG-SGA) [34,35,40,43,44,45]. Two studies used the Mini Nutritional Assessment (MNA) [41,42,63], one used the Global Leadership Initiative on Malnutrition (GLIM) criteria [36,49], one used the Subjective Global Assessment (SGA) [37], and one used the Prognostic Nutritional Index (PNI) [39,64]. Among studies using the PG-SGA (Stage A = well-nourished, Stage B = moderate malnutrition, and Stage C = severe malnutrition), Huang et al. [40] reported a statistically significant improvement in nutrition status following CCRT with an exercise intervention (*p* < 0.001). Wu et al. [43] found that nutrition status remained stable over 12 months in both surgical groups (mean PG-SGA = 2, indicating Stage B). In Yang et al. [35], 63% participants had malnutrition (either Stage B or C) across both groups. Another study by Wu et al. [44] saw improvement in nutrition status between six and twelve months in one surgical group, and it remained stable in the other. Akad et al. [34] reported that there was a proportion of patients (close to 12%) classified as severely malnourished (Stage C) within both groups. Gharagozlian et al. [37] reported that most participants were well-nourished postoperatively, with only one (5%) severely malnourished patient (SGA-C). Similarly, Wu et al. [45] found that participants remained well-nourished at both three and six months post-surgery.

Hsu et al. [41] employed the MNA to evaluate nutrition status and found a statistically significant decline in nutrition status at three months post-treatment compared to pre-surgery (*p* = 0.03), indicating worsening malnutrition. However, by 12 months, nutrition status had improved overall compared to the baseline [41]. In a separate study, the GLIM criteria were used to diagnose malnutrition [36], where the proportion of participants with malnutrition increased from one month post-surgery to twelve months post-surgery (78% to 87%) [36].

### 3.19. Weight Change

Six studies assessed weight change following treatment [37,39,40,41,43,44]. Wu et al. [43] reported more than 10% weight loss at 12 months postoperatively in both surgical groups after proximal laparoscopic gastrectomy. Similarly, in the second study by Wu et al. [44], the mean percentage weight loss at 12 months was also above 10%. Fu et al. [39] observed substantial body weight loss in both groups at one year post-surgery and reported a significant difference between the two surgical groups (*p* = 0.039). One study reported that 45% of participants experienced weight loss exceeding 10% of their preoperative body weight [37]. Hsu et al. [41] observed a statistically significant decrease in weight at three, six, and twelve months post-surgery (*p* < 0.01 at three-, six-, and twelve-month follow-up timepoints). Huang et al. [40] reported stable weight pre- and post-CCRT, with mean weight changing only marginally.

### 3.20. Body Mass Index (BMI)

Five studies reported BMI at follow-up [37,39,43,44,45]. Wu et al. [43] showed slight increases in BMI over time in both surgical groups after proximal laparoscopic gastrectomy. In another study by Wu et al. [44], BMI slightly increased in one surgical group and decreased in another. Gharagozlian et al. [37] reported a decline in BMI following surgery. Fu et al. [39] and Wu et al. [45] found that BMI remained relatively stable at six and twelve months post-surgery.

### 3.21. Muscle Mass

Four studies investigated muscle mass [36,37,40,41], with three using bioelectrical impedance analysis (BIA) [37,40,41,65] and one study using the Ziostation software programme [66]. Huang et al. [40] reported that BIA measures such as appendicular skeletal muscle index, body cell mass, and fat-free mass remained relatively stable, while mean levels of lean body mass decreased slightly. However, phase angle in both arms and legs significantly declined post-treatment (*p* < 0.001) [40]. Hsu et al. [41] reported a significant reduction in skeletal muscle mass at three months post-surgery (*p* = 0.03), followed by recovery at six and twelve months. Gharagozlian et al. [37] found low appendicular skeletal muscle index in both male and female patients postoperatively. Nishibeppu et al. [36] showed that nearly half of the participants had low muscle mass using the Ziostation programme (49%) [66].

### 3.22. Muscle Strength

Muscle strength was assessed in three studies using hand grip strength (HGS) [37,40,41,67]. Two studies reported a statistically significant reduction in HGS following treatment [40,41], whereas Gharagozlian et al. [37] reported that HGS remained within normal ranges post-treatment.

### 3.23. Fat Mass

Two studies assessed fat mass using BIA (32, 33). Huang et al. [40] found that the percentage of body fat remained stable pre- and post-CCRT. In contrast, Hsu et al. [41] reported a significant reduction in visceral fat mass at all three follow-up timepoints post-surgery.

## 4. Discussion

To our knowledge, this is the first systematic review to explore and synthesise the current literature on the presence and effect of nutrition impact symptoms following curative-intent treatment for UGI cancers, including gastric, oesophageal, and pancreatic malignancies. In the included studies, nutrition impact symptoms were highly prevalent post-treatment and were frequently associated with deteriorations in nutrition status. The main nutrition impact symptoms experienced across the different cancers were reflux, abdominal pain, diarrhoea, and constipation, with the prevalence of symptoms measured up to 12 months post-treatment. The absence of studies examining the duration of nutrition impact symptoms and their prolonged effects on nutrition status beyond 12 months limits our understanding of their long-term persistence and ongoing impact on nutrition status and health outcomes. Heterogeneity in study design, data collection timepoints, and methods with which to assess nutrition impact symptoms and nutrition outcome measures, including the various tools used, preclude evaluating the impact of these side effects on clinical outcomes. These findings highlight the need for more consistent approaches to identifying nutrition impact symptoms and ongoing nutrition follow-up to assist in improving QoL and nutrition status in survivors of UGI cancer.

The evidence synthesised in this review demonstrates that nutrition impact symptoms persist for up to 12 months and likely much longer after curative-intent treatment, and likely contribute to deteriorations in nutrition status [34,36,37,41,43,44]. Across the included studies, 12 distinct nutrition impact symptoms were reported with varying prevalence and severity across multiple post-treatment timepoints up to 12 months. This highlights the multidimensional and dynamic nature of post-treatment symptoms, a pattern also observed in other tumour streams. A review on patients with head and neck cancer within 12 months post-treatment indicated that 90% of patients experienced at least one nutrition impact symptom [68]. Common symptoms such as dysphagia, early satiety, and anorexia reduce nutrient assimilation and can lead to cancer-related malnutrition, sarcopenia, and cancer cachexia [15,27]. These conditions can significantly decrease QoL and reduce survival [19]. Recent large-scale data from Australian hospitals highlight that symptoms like dysphagia persist after neoadjuvant treatment, yet limited access and inadequate referral pathways to dietetic support post-treatment were thought to prevent timely intervention [22]. Similarly, a review on nutrition care in gastric cancer noted ongoing post-surgical issues such as PEI, dumping syndrome, and micronutrient deficiencies, but also a lack of nutrition monitoring post-surgery to help manage these [15]. Given that the multifactorial symptom burden is a driver for nutrition decline post-treatment, interventions to manage these symptoms are vital within this patient population to prevent deteriorations in nutrition status.

Given the high prevalence of nutrition impact symptoms and their substantial impact on nutrition status, the early recognition and proactive management of malnutrition in cancer survivors is vital and recognised in evidence-based guidelines [26,69,70] and cancer position statements [24,71]. While these guidelines suggest that nutrition interventions can improve outcomes when initiated early, pathways with which to identify nutrition impact symptoms and those at risk of malnutrition post-treatment remain limited in cancer survivors [15,68]. Although many studies within this review measured nutrition impact symptoms using multi-symptom assessment tools, no studies described targeted management strategies or follow-up care to manage these symptoms. Screening for nutrition risk and symptoms can be completed by any health care professional for appropriate dietetic referral and assessment, in line with current evidence-based practice [24,69,70]. However, the ability for health care professionals to screen for nutrition impact symptoms and nutrition status during the post-treatment phase is limited, likely due to the loss of a primary oncology treatment team [5]. Once in the community, no expert dietetic workforce is available; there are also cost barriers and patients may not realise they need or how to seek support [5,72]. These gaps contribute to missed opportunities for timely nutrition intervention to address nutrition impact symptoms and exacerbate the risk of nutrition decline. Early screening to detect nutrition impact symptoms and manage these symptoms before deteriorations in nutrition status occur may lead to lower risk of complications and improved QoL as well as survival status of cancer survivors [73]. Future research should focus on integrating routine nutrition follow-up into survivorship care to assess persistent nutrition impact symptoms and enhance long-term nutrition outcomes.

Unintentional weight loss occurs when energy intake is less than expenditure, without deliberate effort [74], and often exists when one or more nutrition impact symptoms are present. Studies within this review reported weight loss and limited weight maintenance up to 12 months post-treatment [37,39,43,44]. This highlights that nutrition impact symptoms impair food consumption and nutrient absorption, resulting in nutrition deficits [15,27]. A randomised controlled trial of patients with gastrointestinal or head and neck cancers undergoing radiotherapy found that those who received usual care (standard education from nursing staff, information brochure on nutrition, and some samples of oral nutrition supplements) experienced greater weight loss compared to patients who received targeted nutrition interventions including dietary counselling, oral nutrition supplements, and regular follow-ups [75]. This presents the need for ongoing nutrition interventions in cancer survivors to prevent unintentional weight loss.

Multiple studies reported a high proportion of participants as being malnourished post-treatment [34,36,37,41,43,44], with the majority identifying concurrent nutrition impact symptoms [34,37,39,41,42,43,44,45], demonstrating a link between nutrition impact symptoms and the presence of malnutrition. Importantly, it seems not one isolated nutrition impact symptom but the cumulative burden of multiple gastrointestinal, function, and systemic symptoms such as reflux, diarrhoea, dysphagia, and dumping syndrome that compromises dietary intake, nutrient absorption, and overall nutrition status. These findings suggest that nutrition deterioration is often driven by cumulative symptoms rather than by a single dominant symptom. Understanding this interplay is critical for clinical practice, as it reinforces the need to identify and manage many nutrition impact symptoms in order to prevent reductions in oral intake, nutrition status, and QoL.

The GLIM criteria are a gold-standard validated malnutrition diagnostic tool in clinical practice that incorporate body composition methods used to assess phenotypical aspects that impact nutrition status [49]. Multiple practice guidelines advocate for the use of these criteria because they provide objective insight into muscle mass, strength, and oral intake [24,70,74]. However, very few studies in this review utilised the GLIM criteria, likely due to their relatively recent introduction in 2019. The lack of body composition data in this review is concerning, as most of the included studies reported post-treatment malnutrition without capturing the likely underlying loss of muscle mass and strength. Recent literature highlights that current malnutrition diagnostic tools failing to incorporate body composition may inadequately reflect nutrition status, as a stable body weight can mask substantial muscle depletion [76]. Muscle loss is highly prevalent throughout the cancer journey and is associated with reduced QoL and a poorer prognosis [27]. In the few studies in this review that employed body composition methods, such as BIA, reduced muscle/lean body mass was evident post-treatment [36,37,40,41]. Several studies that incorporated body composition assessment but did not use validated malnutrition diagnostic criteria were excluded from this review. The inconsistent application of validated malnutrition definitions impacts the ability of research findings to be translated into practice. The adoption of body composition methods as part of validated malnutrition diagnostic tools, such as the GLIM criteria [49], will help to identify deteriorations in nutrition status post-treatment and inform future research as well as interventions to mitigate this issue in clinical practice.

Validated nutrition assessment tools are sensitive and specific in identifying most people with malnutrition and require specialised nutrition input and training [16,69]. Unlike surrogate biomarkers (i.e., serum albumin or C-reactive protein) or weight change alone, validated tools evaluate multiple aspects of nutrition status, including dietary intake, weight history, and body composition, allowing for a more comprehensive assessment [49]. Many studies excluded from this review relied on single measures rather than validated tools. International oncology guidelines advocate for the use of validated tools to accurately diagnose malnutrition rather than single measures [16,69]. Many of the excluded studies offered detailed characterisation of nutrition impact symptoms but did not utilise a validated malnutrition diagnostic tool with which to diagnose malnutrition. This highlights a crucial gap in research methodology where tools used to assess and diagnose malnutrition are being underutilised. The future use of validated tools to both describe nutrition impact symptoms and diagnose malnutrition will advance understanding and management in clinical care [69].

To our knowledge, none of the studies included in this review involved a dietitian or qualified nutrition professional as part of the research team. This may be due to a workforce issue across multiple countries, with 1 > 2308 registered dietitians per oncology patient estimated in the United States of America [77,78]. It may also reflect broader under-recognition of the critical role of clinical nutrition in oncological research in some countries [79,80]. Embedding dietitians into research teams will help to improve the use of validated nutrition tools in research and will help to translate evidence into practice. It is recommended that validated tools be used in both clinical practice and research exploring cancer survivorship. This will help to enhance research quality, strengthen evidence, and ultimately improve nutrition outcomes for cancer survivors.

This review had many strengths. A robust and rigorous systematic review methodology was adopted, supported by a comprehensive search strategy that captured a broad range of nutrition impact symptoms and nutrition outcomes across UGI cancers following curative-intent treatment. The systematic review was conducted in accordance with the PRISMA guidelines [29], utilising widely recognised software tools such as EndNote and Covidence [31,33]. By synthesising data from three major cancer types, the findings demonstrated common nutrition challenges that occur post-treatment and their impact on nutrition status.

A key limitation across included studies was the absence of baseline (pre-treatment) data on nutrition impact symptoms and nutrition status in all but three studies [39,40,41]. Without these measures, it is difficult to establish whether symptoms were present before treatment or were induced by therapy. Extensive evidence suggests that nutrition impact symptoms and associated weight loss often occur before diagnosis and worsen during treatment [22,81], making pre-treatment data critical for establishing symptom trajectory and understanding the true nutritional impact of cancer therapy. The lack of baseline data also raises concerns about the potential for selection bias within these studies. It is noteworthy that three out of the eleven studies [43,44,45] were conducted by the same research group, focusing on variations in surgical techniques for gastrectomy. This raises concerns about a potential narrow scope of perspectives and limits the generalisability of findings. The majority of studies were conducted in the Asia–Pacific region (China, Japan, and Taiwan), meaning findings may reflect local patient populations, healthcare systems, and practices, and may not accurately describe the wider population of the world. This review only included studies in the English language, meaning that there may have been valuable studies published in other languages that were excluded. Only one study examined nutrition impact symptoms and nutrition status in patients following pancreatic cancer surgery, which significantly limits our ability to translate these findings to the broader pancreatic cancer population. There was substantial heterogeneity in tools used to identify nutrition impact symptoms across the included studies, with 12 different instruments employed, making comparison and understanding of the true prevalence of symptoms across studies difficult. Some studies relied on single-symptom scales (e.g., FOIS, SNAQ, Fatigue Symptom Inventory, and dysphagia score), while others used more comprehensive tools like PGSAS-45. While single-symptom tools are valid for targeted assessments, they do not fully capture the complexity and dynamic nature of nutrition impact symptoms post-treatment [82]. The studies included in this review tracked patients only up to 12 months post-curative-intent treatment. This is a notable limitation in patient care, screening for nutrition impact symptoms, and guiding dietetic practice given that advancements in medical treatment have resulted in greater survival beyond 12 months post-treatment in gastric and oesophageal cancer populations [83,84]. Addressing these limitations in future research will be essential to better understand the nutrition impact of cancer treatment and inform better patient-centred survivorship care.

This review identifies several key priorities for future research. Most studies within this review failed to assess or prioritise QoL. Future studies should identify and explore QoL to ensure that current and future practice changes positively impact people. Without understanding the effect that nutrition impact symptoms have on QoL in these patients, there is limited ability to be able to manage these symptoms and provide appropriate supportive care. There is a need for comprehensive validated tools that capture multiple symptoms alongside QoL, such as PGSAS-45 or the EORTC-QLQ-30. Implementing these tools will enable more consistent and detailed symptom reporting and facilitate the assessment of symptom trajectories across the cancer care continuum. By reducing heterogeneity in the assessment tools used, this will allow for a greater understanding of the true prevalence of nutrition impact symptoms in future studies and clinical practice. Utilising these multi-symptom tools in future research and clinical practice will allow for more evidence of the burden of nutrition impact symptoms post-treatment and help to inform earlier, more targeted interventions to be implemented.

Dietitians are qualified to provide evidence-based medical nutrition therapy to identify, treat, and manage nutrition impact symptoms and to prevent malnutrition and sarcopenia. Studies suggest a multi-modal rehabilitation approach including dietetic, medical oncology, gastroenterology, and exercise interventions from a physiotherapy or exercise physiologist is vital for cancer survivors to improve nutrition status post-treatment [9,85]. However, long-standing malnutrition and nutrition impact symptoms in patients who have received curative cancer treatment are largely under-recognised and, therefore, under-diagnosed [6].

Future studies should incorporate a baseline assessment of both nutrition impact symptoms and nutrition status using validated tools to better characterise changes over time. This approach will strengthen the understanding of the prevalence and progression of nutrition impact symptoms after curative-intent treatment and help inform strategies with which to improve symptom management and nutrition status across the cancer journey. Combining the use of validated nutrition assessment tools such as GLIM with objective body composition measures such as computed tomography (CT), BIA, or calf circumference will provide a more complete understanding of nutrition status. Integrating these approaches into research and clinical practice will help to detect loss of muscle mass and strength as well as changes in dietary intake and weight. Future trials evaluating the prevalence, severity, and impact of nutrition impact symptoms on nutrition status should include longer follow-up timepoints to evaluate the longevity of nutrition impact symptoms post-treatment. Interventional studies exploring the management of symptoms and the impact this has on nutrition status are encouraged to promote the need for further screening and nutrition management in cancer survivors. It is recommended that future research should be conducted globally across diverse populations and settings to better understand the global prevalence of nutrition impact symptoms post curative-intent treatment in cancer survivors and the impacts on nutrition status. The Nourish Point Prevalence Study (2019–2020) is a valuable contribution to this research field, having assessed nutrition impact symptoms and nutrition status in patients undergoing surgery for UGI cancers using validated methods [22]. This study employed the PG-SGA to diagnose malnutrition and identify nutrition impact symptoms alongside muscle strength measures such as HGS [22]. Consistent with the recommendations of this review, future studies should adopt similar methodologies that integrate validated tools for nutrition status, symptom burden, and body composition. This will enable a more holistic understanding of post-treatment challenges and support the development of timely, targeted interventions.

## 5. Conclusions

This review highlights that nutrition impact symptoms commonly persist over the long term following curative-intent treatment for UGI cancers, contributing to long-term nutrition deterioration, increased morbidity, and reduced survival. As such, changes to current practice are recommended to include nutrition screening, referral, and assessments in survivorship care pathways. Ongoing follow-up should occur post-treatment and prioritise the identification and management of nutrition impact symptoms to prevent deteriorations in nutrition status. Interventions should be evidence-based, multidisciplinary, and sustained beyond the initial treatment period to mitigate their long-term consequences and improve patient outcomes.

## Figures and Tables

**Table 1:** Study characteristics.

Author (Country)	Study Design	Cancer Type	Stage	Treatment	Sample Size	Age	Sex (% Female Total Sample)	Data Collection Timepoints	Intervention Provided	Outcomes of Interest
Akad et al., 2024 [34]	Cross-sectional study	Gastric	I–III	Surgery; total and subtotal gastrectomy	51	66.18 ± 12.93	33.3	‘Post-surgery’ (not described)	Standard nutrition supplements from the hospital	Nutrition status Performance status
Fu et al., 2023 [39]	Retrospective cohort study	Gastric	I–IV	Surgery; two surgical techniques: RG and LG for total gastrectomy	409	RG: 63.34 ± 7.91 LG: 64.08 ± 8.52	29	Preoperative 3 months post-surgery 6 months post-surgery 1 year post-surgery	-	Nutrition status
Gharagozlian et al., 2020 [37]	Cross-sectional study	Gastric	I–III	Surgery; total or subtotal gastrectomy	21	60 ± 12.6	48	28.7 (8.3) months post-surgery	57% consulted a dietician before surgery 86% received dietetic follow-up in the hospital postoperatively	Nutrition status Symptom severity
Huang et al., 2024 [40]	Non-randomised controlled trial	Oesophageal	II–IV	Chemoradiotherapy	67	57 ± 8	3	Baseline 6–8 weeks post-completion of chemoradiotherapy	Exercise programme	Physical fitness Nutrition status Hand grip strength Body composition Functional oral intake
Hsu et al., 2024 [41]	Prospective cohort study	Pancreatic	I–IV	Surgery; distal pancreatectomy and splenectomy, total pancreatectomy, or bypass operation	89	59.87 ± 11.70	49.4	Before surgery 3 months post-surgery 6 months post-surgery 12 months post-surgery	-	Nutrition status Fatigue
Nishibeppu et al., 2024 [36]	Retrospective cohort study	Gastric	I–III	Surgery; distal or total gastrectomy	69	Data group by GLIM criteria: Normal/moderate malnutrition: 65.4 ± 10.0 Severe malnutrition: 66.4 ± 13.3	52	Baseline 1 month post-surgery 1 year post-surgery	CGM monitoring	Glucose fluctuations Nutrition status
Wang et al., 2022 [42]	Cross-sectional study	Gastric	I–III	Surgery; total or subtotal gastrectomy	101	66.5 ± 14.0, range: 25–89	47	Mean: 10.9 ± 7.6 months post-surgery	-	Nutrition status
Wu et al., 2024 [43]	Retrospective cohort study	Gastric	I–II	Surgical techniques: SOA vs. DTA after laparoscopic proximal gastrectomy	43	SOA: 65.5 (60.8, 71.8) DTA: 70 (66, 76) median (range)	16	Baseline 3 months post-surgery 6 months post-surgery 12 months post-surgery	-	Nutrition status
Wu et al., 2024 [44]	Retrospective cohort study	Upper gastric and esophagogastric junction	I–II	Surgery; two surgical techniques; modified Kamikawa anastomosis and DTA after laparoscopic proximal gastrectomy	42	Range: 40–83	19	Baseline 6 months post-surgery 12 months post-surgery	-	Postoperative nutrition status Gastroesophageal reflux
Wu et al., 2024 [45]	Case series	Esophagogastric and gastric	I–II	Surgery; modified Kamikawa anastomosis for laparoscopic proximal gastrectomy	26	68.846 ± 1.352	15	Baseline 6 months post-surgery 12 months post-surgery	-	Symptoms of reflux Nutrition status
Yang et al., 2024 [35]	Retrospective cohort study	Upper gastric	I–II	Surgery; laparoscopic surgery vs. Da Vinci robotic surgery for proximal subtotal gastrectomy	35	Median = 63 Range: 53–72	17	1–42 months post-surgery Median follow-up 24 months	-	Nutrition status

RG, robotic gastrectomy; LG, laparoscopic gastrectomy; GLIM, Global Leadership Initiative on Malnutrition; SOA, side overlap anastomosis; DTA, double-tract anastomosis; QoL, quality of life; and CGM, continuous glucose monitoring.

**Table 2:** Nutrition impact symptoms.

Author	Symptom Tool Used	Symptoms Reported	Baseline	Follow-Up Timepoint 1	Follow-Up Timepoint 2	Follow-Up Timepoint 3
Akad et al., 2024 [34]				Postoperative:		
	ORD 0 = no dysphagia 1 = symptomatic; able to eat regular diet 2 = symptomatic; altered eating/drinking	Dysphagia		Both groups: score 0: 34 (66.7%), score 1: 8 (15.7%), and score 2: 9 (17.6%)		
	SNAQ (lower rating indicates better appetite and reduced risk of malnutrition)	Appetite		Both groups: low: 1 (2%), moderate: 2 (3.9%), and high: 48 (94.1%)		
Fu et al., 2023 [39]	Quality of life questionnaire-stomach 22, qlq-sto22 + EORTC QLQ-30 (higher scores indicate worse condition)		Preoperative (RG vs. LG):	3 months post-surgery (RG vs. LG):	6 months post-surgery (RG vs. LG):	12 months post-surgery (RG vs. LG):
		Dysphagia ^a^	10.3 ± 6.3 vs. 10.9 ± 5.7	27.8 ± 12.3 vs. 30.9 ± 10.8	16.4 ± 6.7 vs. 18.4 ± 9.7	12.3 ± 3.1 vs. 13.1 ± 2.9
		Sour regurgitation ^a^	9.2 ± 7.1 vs. 8.9 ± 6.5	10.3 ± 6.3 vs. 12.4 ± 7.1	8.2 ± 4.3 vs. 8.6 ± 3.7	8.1 ± 3.3 vs. 8.3 ± 3.7
		Belching ^a^	5.1 ± 4.2 vs. 5.4 ± 3.9	11.2 ± 6.1 vs. 14.1 ± 8.7 ↑	7.1 ± 3.9 vs. 9.4 ± 4.5 ↑	6.2 ± 2.2 vs. 6.7 ± 2.4
		Abdominal pain ^a^	8.1 ± 4.9 vs. 8.3 ± 4.1	15.7 ± 6.8 vs. 19.1 ± 7.3 ↑	12.3 ± 4.9 vs. 14.4 ± 5.7 ↑	9.3 ± 3.6 vs. 10.6 ± 3.3 ↑
		Diarrhoea ^a^	6.9 ± 5.1 vs. 7.1 ± 5.2	13.4 ± 7.1 vs. 16.6 ± 8.7 ↑	12.9 ± 5.3 vs. 14.1 ± 4.9	9.9 ± 4.7 vs. 10.1 ± 4.6
		Fatigue ^a^	7.1 ± 4.7 vs. 7.3 ± 4.4	18.7 ± 10.3 vs. 21.2 ± 9.8	11.3 ± 5.2 vs. 13.7 ± 6.5 ↑	8.4 ± 3.9 vs. 9.2 ± 3.1
	Correlation between nutrition status (using PG-SGA scoring) and symptoms (using QLQ-STO22 and QLQ-C30) (PG-SGA has a continuous score from 0 to 16, where higher scores indicate higher malnutrition risk)	Dysphagia ^b^				0–3 = 14.4 ± 6.1 4–8 = 15.2 ± 7.3 ≥ 9 = 17.8 ± 9.2 ↑
		Sour regurgitation ^b^				0–3 = 7.2 ± 4.1 4–8 = 6.9 ± 3.4 ≥9 = 7.7 ± 5.4 ↑
		Belching ^b^				0–3 = 8.4 ± 5.3 4–8 = 8.2 ± 4.6 ≥ 9 = 8.9 ± 3.8 ↑
		Abdominal pain ^b^				0–3 = 16.3 ± 8.2 4–8 =19.4 ± 7.7 ≥9 = 22.6 ± 6.3 ↑
		Diarrhoea ^b^				0–3 = 7.2 ± 5.2 4–8 = 9.3 ± 4.9 ≥ 9 = 12.2 ± 6.4 ↑
		Fatigue ^b^				0–3 =21.1 ± 9.3 4–8 =22.5 ± 13.2 ≥9 = 22.9 ± 12.6 ↑
Gharagozlian et al., 2020 [37]	GSRS syndrome (7-point Likert scale, where higher scores indicate worse conditions)			Post-surgery: 28.7 (8.3) months		
		Abdominal pain ^a^		Well nourished = 2.0 (0.88) Malnourished = 2.9 (0.72) ↑		
		Diarrhoea ^a^		Well nourished = 2.3 (1.5) Malnourished = 2.6 (1.3)		
		Constipation ^a^		Well nourished = 1.8 (0.84) Malnourished = 2.9 (1.4)		
		Indigestion ^a^		Well nourished = 2.9 (1.0) Malnourished = 3.5 (0.43)		
		Reflux ^a^		Well nourished = 1.5 (0.97) Malnourished = 2.3 (1.4)		
	SF-36 scale (score out of 100, where higher scores indicate better conditions)	Bodily pain ^a^		Well nourished = 79.2 (22.0) Malnourished = 47.6 (13.7) ↑		
Huang et al., 2024 [40]	FOIS ^b^ (7-point Likert scale, 0 = NBM and 7 = oral intake with no restrictions)	Dysphagia ^b^	Pre-CCRT:	Post-CCRT (6–8 weeks):		
			5.5 ± 1.7	5.7 ± 1.6		
Hsu et al., 2024 [41]	Fatigue Symptom Inventory (higher scores indicate higher level of fatigue, ranging from 0 to 127 points)	Fatigue ^b^	Before surgery:	3 months:	6 months:	12 months:
			18.57 ± 22.50	21.91 ± 23.88	16.31 ± 21.26	16.42 ± 20.81
Nishibeppu, et al., 2024 [36]	PGSAS-37 (7-point Likert scale, where higher scores indicate worse conditions)			1 month (normal/moderate malnutrition vs. severe):	1 year (normal/moderate malnutrition vs. severe):	
		Oesophageal reflux ^a^		2.0 ± 0.9 vs. 1.99 ± 0.9	1.7 ± 1.0 vs. 1.7 ± 0.6	
		Abdominal pain ^a^		2.1 ± 0.8 vs. 2.2 ± 0.9	1.5 ± 0.7 vs. 1.5 ± 0.7	
		Indigestion ^a^		2.2 ± 0.8 vs. 2.0 ± 0.7	2.2 ± 0.9 vs. 2.2 ± 1.0	
		Diarrhoea ^a^		1.7 ± 0.7 vs. 2.1 ± 1.1	1.8 ± 0.7 vs. 2.6 ± 1.1 ↑	
		Constipation ^a^		2.2 ± 0.9 vs. 2.5 ± 1.1	2.2 ± 0.9 vs. 2.1 ± 1.0	
		Dumping ^a^		1.8 ± 1.0 vs. 2.0 ± 1.2	1.5 ± 0.9 vs. 2.1 ± 1.2 ↑	
Wang et al., 2022 [42]	Gastric Cancer Subscale of the FACT-Ga. (4-point Likert scale—0, not at all; 4, very much)			Post-surgery:		
		Being bothered by gas (flatulence)		1.31 ± 1.34 0–4		
		Having stomach problems that worry me		1.05 ± 1.13 0–4		
		Having fullness or heaviness in the stomach		1.01 ± 1.09 0–4		
		Having discomfort or pain when eating		0.94 ± 1.06 0–4		
		Feeling tired		0.93 ± 1.11 0–4		
		Having swelling or cramps in the stomach area		0.88 ± 1.12 0–4		
		Having discomfort or pain in the stomach area		0.89 ± 1.02 0–4		
		Bothered by reflux or heartburn		0.72 ± 1.04 0–4		
		Losing weight		0.40 ± 0.86 0–4		
		Loss of appetite		0.66 ± 1.09 0–4		
		Having trouble swallowing food		0.28 ± 0.74 0–4		
		Having diarrhoea		0.49 ± 0.84 0–4		
		Feeling weak all over		0.63 ± 1.06 0–4		
Wu et al., 2024 [43]	PGSAS-45 (7-point Likert scale, where higher scores indicate worse conditions)			12 months (SOA vs. DTA):		
		Oesophageal reflux subscale ^a^		3.0 ± 1.2 vs. 4.1 ± 1.3 ↑		
		Abdominal pain subscale ^a^		1.7 (1.3, 3.0) vs. 2.0 (1.3, 3.3)		
		Indigestion subscale ^a^		2.3 (2.3, 3.0) vs. 2.5 (2.3, 3.5)		
		Diarrhoea subscale ^a^		1.3 (1.3, 2.0) vs. 1.3 (1.7, 2.0)		
		Constipation subscale ^a^		1.3 (1.3, 1.7) vs. 1.3 (1.3, 1.7)		
		Dumping subscale ^a^		1.3 (1.3, 1.3) vs. 1.3 (1.3, 1.3)		
		Increased flatus ^a^		2.5 (2, 3) vs. 4 (3, 5) ↑		
		Loose stools ^a^		1 (1, 2) vs. 1 (1, 2)		
	Los Angeles Scale	Reflux oesophagitis ^a^		2 vs. 9 ↑		
		Grade A		2 vs. 5		
		Grade B		0 vs. 4		
Wu et al., 2024 [44]	PGSAS-45 (7-point Likert scale, where higher scores indicate worse conditions) GERD scale score Los Angeles Scale (1–4, where higher scores indicate worse conditions)			6 months (modified Kamikawa vs. DTA):	12 months (modified Kamikawa vs. DTA):	
		Oesophageal reflux subscale ^a^			3.1 ± 1.3 vs. 4.0 ± 1.3	
		Abdominal pain subscale ^a^			1.7 (1.3, 4.3) vs. 2.0 (1.3, 4.3)	
		Indigestion subscale ^a^			2.4 (2.0, 4.0) vs. 2.5 (2.0, 4.8)	
		Diarrhoea subscale ^a^			1.3 (1.0, 2.7) vs. 1.5 (1.0, 2.7)	
		Constipation subscale ^a^			1.3 (1.0, 2.3) vs. 1.3 (1.0, 2.3)	
		Dumping subscale ^a^			1.3 (1.0, 2.3) vs. 1.3 (1.0, 2.3)	
		Increased flatus ^a^			3.0 (1.0, 6.0) vs. 3.5 (1.0, 6.0)	
		Loose stools ^a^			1.0 (1.0, 2.0) vs. 1.0 (1.0, 2.0)	
		Gastroesophageal reflux disease ^a^		3.0 (2.0–4.0) vs. 3.0 (2.0–4.0)	3.0 (2.0–4.0) vs. 2.5 (2.0–4.0)	
		Grade B reflux esophagitis ^a^			1 vs. 2	
Wu et al., 2024 [45]			6 months:	12 months:		
	GERD scale	Gastroesophageal reflux disease	3 (2–4)	3 (2–4)		
	Los Angeles Scale	Reflux esophagitis	0			
Yang et al., 2024 [35]	-	Reflux esophagitis		Postoperative: (laparoscopic vs. Da Vinci robotic esophagogastric anastomosis):		
				1 case of reflux in the laparoscopic surgery group. Nil in the Da Vinci robotic group		

ORD, observer-reported dysphagia; SNAQ, Simplified Nutritional Appetite Questionnaire; RG, robotic gastrectomy; LG, laparoscopic gastrectomy; PG-SGA, Patient-Generated Subjective Global Assessment; EORTC, European Organisation for Research and Treatment of Cancer; GSRS, Gastrointestinal Symptom Rating Scale; SF-36, 36-Item Short Form Health Survey; FOIS, Functional Oral Intake Scale; PGSAS-37, Postgastrectomy Syndrome Assessment Scale-37; FACT-GA, Functional Assessment of Cancer Therapy-Gastric; PGSAS-45, Postgastrectomy Syndrome Assessment Scale-45; SOA, side overlap anastomosis; DTA, double-tract anastomosis; and GERD, gastroesophageal reflux disease. Legend: ^a^ indicates a between-group analysis. ^b^ indicates a within-group analysis. ↑ = significant increase in reported outcome measure. If there is no significant symbol (i.e., ↑ or ↓), then there is no significant between-group change in outcome. [34]’s outcomes are presented as n (%). [39]’s outcomes are presented as mean ± SD. [37]’s outcomes are presented as mean (SD). [40]’s outcomes are presented as mean ± SD. [41]’s outcomes are presented as mean ± SD. [36]’s outcomes are present as mean ± SD. [42]’s outcomes are presented as mean ± SD, mean (range), and mean. [43]’s outcomes are presented as mean ± SD, mean (range), and mean. [44]’s outcomes are presented as mean (range). [45]’s outcomes are presented as mean (range). [35]’s outcomes are presented as n.

**Table 3:** Nutrition outcome measures reported within the included studies.

Author	Malnutrition Risk		Malnutrition Assessment			Body Composition			
	Tool	Score	Tool	Score	Weight (kg)	BMI (kg/m^2^) (Mean ± SD)	Muscle/Lean Body Mass	Fat Mass	Muscle Strength
Akad et al., 2024 [34]	NRS-2002 Score < 3—no nutrition risk; >3—nutrition risk (n (%))	Both groups:	PG-SGA (n (%)) (Stage A = well nourished Stage B = suspected or moderate malnutrition Stage C = severe malnutrition)	Both groups:	-	-	-	-	
		Score 0: 44 (88.2%) Score 1: 1 (2%)							
				Stage A: 15 (29.4%)					
				Stage B: 30 (58.8%)					
		Score 2: 3 (5.9%)		Stage C: 6 (11.8%)					
		Score 3: 2 (3.9%)							
Fu et al., 2023 [39]	NRS-2002 Score < 3—no nutrition risk; >3—nutrition risk (n (%))	(RG vs. LG)	PNI (higher scores indicate better nutrition)	(RG vs. LG)	(RG vs. LG)	(RG vs. LG)	-	-	
		Score < 3 Entire cohort: 69 (65.1) vs. 179 (59.1)							
				Preoperative ^a^: 422.7 ± 75.3 vs. 437.02 ± 81.2	Preoperative ^a^: 63.2 ± 9.7 vs. 62.9 ± 9.4	Preoperative ^a^: 24.69 ± 4.01 vs. 25.15 ± 3.14			
		Score ≥ 3 Entire cohort: 37 (34.9) vs. 124 (40.9)		3 months ^a^: 362.1 ± 61.4 vs. 369.5 ± 57.6	3 months ^a^: 57.8 ± 6.5 vs. 56.1 ± 6.1				
				6 months ^a^: 370.4 ± 53.5 vs. 373.6 ± 55.8	6 months ^a^: 58.2 ± 6.8 vs. 57.3 ± 6.3				
				1 year ^a^: 379.5 ± 51.2 vs. 383.4 ± 51.4	1 year ^a^: 58.6 ± 7.2 vs. 57.8 ± 6.9				
Gharagozlian et al., 2020 [37]	-	-	SGA (n (%)) (SGA-A = well nourished SGA-B = suspected or moderate malnutrition) SGA-C = severe malnutrition)	SGA-A = 15 (72%)	Weight loss (%): 12.8 ± 11.6	Preoperative BMI: 26.0 ± 4.8	BIA: (mean ±SD)	-	HGS:
				SGA-B = 5 (24%)	> 10% loss of current weight: 9 (45%)	Postoperative BMI: 22.2 ± 3.3	ASMI: Females (kg/m^2^) = 3.2 ± 0.60 Males (kg/m^2^): 4.4 ± 0.51 (low scores are ≤7.0 kg/m^2^ in men and ≤5.5 kg/m^2^ in women)		Females (kg) = 23.6 ± 5.5 Males (kg) = 43.1 ± 9.3 (low strength defined as <27 kg for males and <16 kg for females)
				SGA-C = 1 (5%)					

									EWGSOP: (n (%)) Pre-sarcopenia: 20 (100.0) Sarcopenia: 1 (5.0)
Huang et al., 2024 [40]	-	-	PG-SGA score ^b^ (mean ± SD) (higher scores indicate greater severity of malnutrition)	Pre-CCRT: 6.2 ± 3.3	Pre-CCRT ^b^: 65.5 ± 12.6	-	BIA: (pre-CCRT vs. post CCRT)	BF (%) ^b^: (pre-CCRT vs. post-CCRT) 23.0 ± 6.7 vs. 22.3 ± 6.4	HGS (kg) ^b^: 41.7 ± 7.9 vs. 39.6 ± 8.6 ↓
				6–8 weeks post-CCRT: 3.6 ± 3.3 ↓ (improved nutrition status)	Post-CCRT ^b^: 65.4 ± 11.9				
							ASMI ^b^ (kg/m^2^): 7.63 ± 0.97 vs. 7.64 ± 1.04		
							BCM (kg) ^b^: 32.7 ± 5.5 vs. 32.6 ± 5.7		
							FFM (kg) ^b^: 49.9 ± 8.6 vs. 50.4 ± 8.6		
							LBM (kg) ^b^: 46.9 ± 8.8 vs. 47.8 ± 8.2		
							PARA ^b^: 6.0 ± 0.8 vs. 5.5 ± 0.8 ↓		
							PALA ^b^: 5.9 ± 0.8 vs. 5.5 ± 0.9 ↓		
							PATR ^b^: 8.6 ± 1.7 vs. 8.0 ± 1.5 ↓		
							PARL ^b^: 6.2 ± 0.9 vs. 5.6 ± 1.0 ↓		
							PALL ^b^: 5.9 ± 0.9 vs. 5.4 ± 1.0 ↓		
Hsu et al., 2024 [41]	-	-	MNA (mean ± SD) (lower scores indicate poorer nutrition status, with a range from 0 to 30)				BIA: skeletal muscle mass (mean ± SD):	BIA: visceral fat mass (mean ± SD):	HGS:
				T0: 23.85 ± 3.63	T0: 60.46 ± 11.44		T0: 21.35 ± 5.71	T0: 2.30 ± 1.37	T0: 26.13 ± 9.25

				T1 ^b^: 22.96 ± 3.37, T1/T0 ↓	T1 ^b^: 57.62 ± 10.69 ↓		T1 ^b^: 21.94 ± 6.03, T1/T0 ↓	T1 ^b^: 1.70 ± 1.13, T1/T0 ↓	T1 ^b^: 23.20 ± 9.07, T1/T0 ↓
				T2 ^b^: 24.59 ± 3.01, T2/T0	T2 ^b^: 56.81 ± 10.71 ↓		T2 ^b^: 22.15 ± 5.52, T2/T0	T2 ^b^: 1.77 ± 1.20, T2/T0 ↓	T2 ^b^: 24.44 ± 9.99, T2/T0 ↓
				T3 ^b^: 25.09 ± 3.57, T3/T0	T3 ^b^: 57.58 ± 11.46 ↓		T3 ^b^: 21.91 ± 4.75 T3/T0	T3 ^b^: 1.87 ± 1.24, T3/T0 ↓	T3 ^b^: 24.10 ± 9.56 T3/T0 ↓
				T0 = before surgery T1 = 3 months after surgery T2 = 6 months after surgery T3 = 12 months after surgery				Lower scores indicate lower fat mass	30 s sit-to-stand test Lower-limb strength:
									T0: 18.46 ± 6.63
									T1 ^b^: 19.15 ± 7.69, T1/T0
									T2 ^b^: 20.54 ± 9.04, T2/T0
									T3 ^b^: 20.44 ± 7.43 T3/T0 ↑ Lower scores indicate lower strength
Nishibeppu et al., 2024 [36]	-	-	GLIM (n)	1 month: Severe: 30 Moderate: 24 No malnutrition: 15	-	Preoperative BMI:	Psoas muscle mass index (PMI) from CT scan: High PMI: 34 participants Low PMI: 35 participants	-	
						GLIM normal/moderate: 23.7 ± 2.6			
						Severe: 20.4 ± 2.8 ↓			
				1 year: Severe: 25 Moderate: 35 No malnutrition: 9					
Wang et al., 2022 [42]	-	-	MNA (n) (lower scores indicate poorer nutrition status, with a range from 0 to 30)	48 = score > 24, indicating well-nourished	-	-	-	-	
				44 = score between 17.5 and 23, indicating risk of malnutrition					
				9 = score < 17, suggesting malnutrition					
Wu et al., 2024 [43]	NRS 2002 (mean (range)) Score < 3—no nutrition risk; >3—nutrition risk	3 months ^a^ SOA: 2 (1.25, 2) DTA: 2 (1, 2)	PG-SGA (mean (range)) (Stage A (1) = well nourished Stage B (2) = suspected or moderate malnutrition Stage C (3) = severe malnutrition)	3 months ^a^: SOA: 2 (1.25, 2) DTA: 2 (1, 2)	12 months ^a^: (%) change: SOA: 12.1 ± 4.6 DTA: 12.9 ± 4.3	3 months ^a^: SOA: 21.3 ± 2.6 DTA: 21.5 ± 2.6	-	-	
		6 months ^a^: SOA: 2 (2, 2) DTA: 2 (1, 2)		6 months ^a^: SOA: 2 (1, 2) DTA: 2 (1, 2)		6 months ^a^: SOA: 21.5 ± 2.9 DTA: 21.4 ± 2.6			
		12 months ^a^: SOA: 1.5 (1, 2) DTA: 1.5 (1, 2)		12 months ^a^: SOA: 2 (1, 2) DTA: 2 (1, 2)		12 months ^a^: SOA: 22.0 ± 2.5 DTA: 22.3 ± 2.6			
Wu et al., 2024 [44]	MUST (0 = low, 1 = moderate, and 2 = high) (mean (range))	6 months ^a^ Modified Kamikawa anastomosis: 1 (1.0–2.0) DTA: 1 (1.0–2.0)	PG-SGA (mean (range)) (Stage A (1) = well nourished Stage B (2) = suspected or moderate malnutrition Stage C (3) = severe malnutrition)	6 months ^a^ Modified Kamikawa anastomosis: 2 (1.0–3.0) DTA: 2 (1.0–3.0)	12 months ^a^: Δ % weight loss Modified Kamikawa anastomosis: 12.6 ± 4.6 DTA: 13.8 ± 5.1	Baseline: Modified Kamikawa anastomosis: 22.2 ± 2. DTA: 21.2 ± 3.3	-	-	
		12 months ^a^ Modified Kamikawa anastomosis: 1 (1.0–2.0) DTA: 1 (1.0–2.0)		12 months ^a^ Modified Kamikawa anastomosis: 2 (1.0–3.0) DTA: 1.5 (1.0–3.0)		6 months ^a^ Modified Kamikawa anastomosis: 22.2 ± 2.7 DTA: 22.8 ± 2.9			
						12 months ^a^ Modified Kamikawa anastomosis: 22.4 ± 2.5 DTA: 21.5 ± 2.9			
Wu et al., 2024 [45]	NRS 2002 Score < 3—no nutrition risk; > 3—nutrition risk (mean (range))	Preoperative: 2 (1–2)	PG-SGA (mean, (range)) (Stage A (1) = well nourished Stage B (2) = suspected or moderate malnutrition Stage C (3) = severe malnutrition)	Preoperative: 1 (1–3)	-	Baseline: 22.6 ± 3.1	-	-	
		6 months: 2 (1–2)		6 months: 1 (1–3)		6 months: 22.6 ± 3.1			
						12 months: 22.6 ± 3.2			
		12 months: 2 (1–2)		12 months: 1 (1–3)					
Yang et al., 2024 [35]	NRS2002 Score < 3—no nutrition risk; > 3—nutrition risk (n (%))	Both groups Combined ^a^:	PG-SGA (n (%)) (Stage A (1) = well nourished Stage B (2) = suspected or moderate malnutrition Stage C (3) = severe malnutrition)	Both groups combined ^a^:	-	Baseline: Laparoscopic: 23.63 ± 2.59 Da Vinci Robot surgery 23.11 ± 2.65	-	-	
		Score 1: 18 (51%)		Score 1: 13 (37%)					
				Score 2: 14 (40%)					
				Score 3: 8 (23%)					
		Score 2: 17 (49%)							

NRS, nutritional risk screening; PG-SGA, Patient-Generated Subjective Global Assessment; RG, robotic gastrectomy, LG, laparoscopic gastrectomy; PNI; Prognostic Nutritional Index; SGA, Subjective Global Assessment; BMI, body mass index; BIA, bioelectrical impedance analysis; ASMI, appendicular skeletal muscle index; HGS, hand grip strength; EWGSOP, European Working Group on Sarcopenia in Older People; CCRT, concurrent chemoradiotherapy; BCM, body cell mass; FFM, fat-free mass; LBM, lean body mass; PARA, phase angle right arm; PALA, phase angle left arm; PATR, phase angle trunk; PARL, phase angle right leg; PALL, phase angle leg left leg; BF, body fat percentage; MNA, Mini Nutritional Assessment; GLIM, Global Leadership Initiative on Malnutrition; PMI, psoas muscle index; CT, computed tomography; SOA, side overlap anastomosis; DTA, double-tract anastomosis; and MUST, Malnutrition Universal Screening Tool. Legend: ^a^ indicates a between-group analysis. ^b^ indicates a within-group analysis. ↑ = significant increase in reported outcome measure. ↓ = significant decrease in outcome measure. If no significant symbol (i.e., ↑ or ↓), there is no significant between-group change in outcome.

## Data Availability

Not applicable.

## Supplementary Materials

The following supporting information can be downloaded at: https://www.mdpi.com/article/10.3390/nu17162691/s1, Supplementary File S1, Search Strategy; Supplementary File S2; SWiM Checklist.

## Abbreviations

The following abbreviations are used in this manuscript:

UGI	Upper gastrointestinal tract
QoL	Quality of life
PEI	Pancreatic exocrine insufficiency
BMI	Body mass index
PRISMA	Preferred Reporting Items for Systematic Reviews and Meta-Analysis
SWiM	Synthesis Without Meta-analysis
PGSAS-45	Postgastrectomy Syndrome Assessment Scale-45
GLIM	Global Leadership Initiative on Malnutrition
PGSAS-37	Postgastrectomy Syndrome Assessment Scale-37
GERD	Gastroesophageal reflux disease
FACT-Ga	Functional Assessment of Cancer Therapy-Gastric
GSRS	Gastrointestinal Symptom Rating Scale
SGA	Subjective Global Assessment
RG	Robotic gastrectomy
LG	Laparoscopic gastrectomy
PG-SGA	Patient-Generated Subjective Global Assessment
EORTC	European Organisation for Research and Treatment of Cancer
FOIS	Functional Oral Intake Scale
SOA	Side overlap anastomosis
DTA	Double-tract anastomosis
SNAQ	Simplified Nutritional Appetite Questionnaire
SF-36	Short Form 36 Health Survey Questionnaire
CCRT	Concurrent chemoradiotherapy
NRS	Nutritional Risk Screening
MUST	Malnutrition Universal Screening tool
MNA	Mini Nutritional Assessment
PNI	Prognostic Nutritional Index
BIA	Bioelectrical impedance analysis
HGS	Hand grip strength
CT	Computed tomography
CGM	Continuous glucose monitoring
ORD	Observer-reported dysphagia
ASMI	Appendicular skeletal muscle index
BCM	Body cell mass
FFM	Fat free mass
LBM	Lean body mass
PARA	Phase angle right arm
PALA	Phase angle left arm
PATR	Phase angle trunk
PARL	Phase angle right leg
PALL	Phase angle left leg
BF	Body fat percentage
EWGSOP	European Working Group on Sarcopenia in Older People
PMI	Psoas muscle index
