# Supplementary material for: The Effect of Nutrition Impact Symptoms on Nutrition Status After Completion of Curative-Intent Treatment for Gastric, Oesophageal, and Pancreatic Cancer: A Systematic Review
Source: Nutrients. 2025 Aug 20;17(16):2691. doi: 10.3390/nu17162691 (PMC12389404; doi:10.3390/nu17162691)
Supplement: Supplementary file 1 [file nutrients-17-02691-s001.zip › Supplementary File S1 - Search Strategy.pdf]

Ovid EMBASE

1 exp esophagus tumor/
2 exp stomach tumor/
3 exp pancreas tumor/
4 (oesophag* adj3 (cancer* or tumo?r* or neoplasm* or carcinoma* or malignan* or adenocarcin*)).mp.
5 (esophag* adj3 (cancer* or tumo?r* or neoplasm* or carcinoma* or malignan* or adenocarcin*)).mp.
6 (stomach adj3 (cancer* or tumo?r* or neoplasm* or carcinoma* or malignan* or adenocarcin*)).mp.
7 (gastric adj3 (cancer* or tumo?r* or neoplasm* or carcinoma* or malignan* or adenocarcin*)).mp.
8 (pancrea* adj3 (cancer* or tumo?r* or neoplasm* or carcinoma* or malignan* or adenocarcin*)).mp.
9 1 or 2 or 3 or 4 or 5 or 6 or 7 or 8
10 exp Chemotherapy/
11 exp Radiotherapy/
12 exp Antineoplastic Agent/
13 exp Surgery/
14 antineoplastic agen*.mp.
15 chemotherap*.mp.
16 chemoradiotherap*.mp.
17 chemo* radiotherap*.mp.
18 chemo radio therap*.mp.

19 radiotherap*.mp.
20 surger*.mp.
21 operat*.mp.
22 radio* therap*.mp.
23 chemo* therap*.mp.
24 immun*therap*.mp.
25 neo adjuvant*.mp.
26 adjuvant treat*.mp.
27 radiation therap*.mp.
28 radiotherapeutic technique*.mp.
29 radio therapeutic technique*.mp.
30 anti neoplastic agen*.mp.
31 anticancer drug*.mp.
32 anti cancer drug*.mp.
33 anticancer agen*.mp.
34 anti cancer agen*.mp.
35 antitumo?r drug*.mp.
36 antitumo?r agen*.mp.
37 anti tumo?r agen*.mp.
38 tumo?r inhibitor.mp.
39 anti tumo?r drug*.mp.
40 10 or 11 or 12 or 13 or 14 or 15 or 16 or 17 or 18 or 19 or 20 or 21 or 22 or 23 or 24 or 25 or 26 or 27 or 28 or 29 or 30 or 31 or 32 or 33 or 34 or 35 or 36 or 37 or 38 or 39
41 gastrointestinal symptom/
42 exp dysphagia/
43 anorexia/
44 exp Nausea/ and vomiting.mp.
45 mucosa inflammation/

46 exp Diarrhea/
47 exp Constipation/
48 exp Fatigue/
49 exp Pain/
50 decreased appetite/
51 decreased appetite.mp.
52 loss of appetite.mp.
53 lack of appetite.mp.
54 anorexi*.mp.
55 nause*.mp.
56 vomit*.mp.
57 emesis.mp.
58 hyperemesis.mp.
59 diarrh?ea.mp.
60 (loose adj1 motion*).mp.
61 constipat*.mp.
62 mucositis.mp.
63 mucosa* inflamm*.mp.
64 early satiety.mp.
65 dysgeusi*.mp.
66 dysphagi*.mp.
67 problem* swallow*.mp.
68 swallow* problem*.mp.
69 (difficul* adj1 swallow*).mp.
70 (dis* adj1 swallow*).mp.
71 unsafe swallow*.mp.
72 deglutition disorder*.mp.

73 dry mouth.mp.
74 symptom*.mp.
75 taste change*.mp.
76 pain*.mp.
77 bloat*.mp.
78 reflux.mp.
79 flatulence.mp.
80 tired*.mp.
81 fatigue*.mp.
82 cancer related fatigue.mp.
83 physical fatigue.mp.
84 mental fatigue.mp.
85 dump* syndrome.mp.
86 nutriti* impact symptom*.mp.
87 epigastric pain.mp.
88 epi gastric pain.mp.
89 hyp?glyc?emi*.mp.
90 pancrea* exocrine insufficien*.mp.
91 discomfort.mp.
92 41 or 42 or 43 or 44 or 45 or 46 or 47 or 48 or 49 or 50 or 51 or 52 or 53 or 54 or 55 or 56 or 57 or 58 or 59 or 60 or 61 or 62 or 63 or 64 or 65 or 66 or 67 or 68 or 69 or 70 or 71 or 72 or 73 or 74 or 75 or 76 or 77 or 78 or 79 or 80 or 81 or 82 or 83 or 84 or 85 or 86 or 87 or 88 or 89 or 90 or 91
93 exp body composition/
94 exp skeletal muscle/
95 exp muscle atrophy/
96 exp Nutritional Status/
97 exp Malnutrition/
98 exp nutritional deficiency/

99	sarcop?en*.mp.
100	body composition.mp.
101	musc* atrophy.mp.
102	musc* mass.mp.
103	musc* wast*.mp.
104	skeletal muscle.mp.
105	nutrition* status.mp.
106	malnutrition.mp.
107	protein energy malnutrition.mp.
108	under nutrition.mp.
109	under nourish*.mp.
110	cachexia.mp.
111	fat free mass.mp.
112	weight los*.mp.
113	lean mass*.mp.
114	malnourish*.mp.
115	undernourish*.mp.
116	cachectic.mp.

117 under weight.mp.

118 underweight.mp.

119 cancer cachexia.mp.

120 93 or 94 or 95 or 96 or 97 or 98 or 99 or 100 or 101 or 102 or 103 or 104 or 105 or 106 or 107 or 108 or 109 or 110 or 111 or 112 or 113 or 114 or 115 or 116 or 117 or 118 or 119

121 9 and 40 and 92 and 120

122 limit 121 to yr="2020 -Current"

<u>Ovid MEDLINE</u>

1 exp Esophageal Neoplasms/

2 Stomach Neoplasms/

3 exp Pancreatic Neoplasms/

4 (stomach adj3 (cancer* or tumo?r* or neoplasm* or carcinoma* or malignan* or adenocarcin*)).mp.

5 (gastr* adj3 (cancer* or tumo?r* or neoplasm* or carcinoma* or malignan* or adenocarcin*)).mp.

6 (oesophag* adj3 (cancer* or tumo?r* or neoplasm* or carcinoma* or malignan* or adenocarcin*)).mp.

7 (esophag* adj3 (cancer* or tumo?r* or neoplasm* or carcinoma* or malignan* or adenocarcin*)).mp.

8 (pancrea* adj3 (cancer* or tumo?r* or neoplasm* or carcinoma* or malignan* or adenocarcin*)).mp.

9 1 or 2 or 3 or 4 or 5 or 6 or 7 or 8

10 exp Chemotherapy, Adjuvant/

11 exp Radiotherapy/

12 General Surgery/

13 exp Antineoplastic Agents/

14 exp Chemoradiotherapy/

15 antineoplastic agen*.mp.

16 chemotherap*.mp.

17 chemoradiotherap*.mp.

18 chemo* radiotherap*.mp.

19	chemo radio therap*.mp.
20	radiotherap*.mp.
21	surger*.mp.
22	operat*.mp.
23	radio* therap*.mp.
24	chemo* therap*.mp.
25	immun*therap*.mp.
26	neo adjuvant*.mp.
27	adjuvant treat*.mp.
28	radiation therap*.mp.
29	radiotherapeutic technique*.mp.
30	radio therapeutic technique*.mp.
31	anti neoplastic agen*.mp.
32	anticancer drug*.mp.
33	anti cancer drug*.mp.
34	anticancer agen*.mp.
35	anti cancer agen*.mp.
36	antitumo?r drug*.mp.
37	antitumo?r agen*.mp.
38	anti tumo?r agen*.mp.
39	tumo?r inhibitor*.mp.
40	anti tumo?r drug*.mp.
41	10 or 11 or 12 or 13 or 14 or 15 or 16 or 17 or 18 or 19 or 20 or 21 or 22 or 23 or 24 or 25 or 26 or 27 or 28 or 29 or 30 or 31 or 32 or 33 or 34 or 35 or 36 or 37 or 38 or 39 or 40
42	exp "signs and symptoms, digestive"/
43	exp Deglutition Disorders/
44	Mucositis/
45	exp Fatigue/

46 exp Pain/
47 decreased appetite*.mp.
48 loss of appetite.mp.
49 lack of appetite.mp.
50 anorexi*.mp.
51 nause*.mp.
52 vomit*.mp.
53 emesis.mp.
54 hyperemesis.mp.
55 diarrh?ea.mp.
56 (loose adj1 motion*).mp.
57 constipat*.mp.
58 mucositi*.mp.
59 mucosa* inflamm*.mp.
60 early satiety.mp.
61 dysgeusi*.mp.
62 dysphagi*.mp.
63 problem* swallow*.mp.
64 swallow* problem*.mp.
65 swallow* problem*.mp.
66 (difficul* adj1 swallow*).mp.
67 (dis* adj1 swallow*).mp.
68 unsafe swallow*.mp.
69 deglutition disorder*.mp.
70 dry mouth.mp.
71 symptom*.mp.
72 taste change*.mp.

73 pain*.mp.

74 bloat*.mp.

75 reflux.mp.

76 flatulence.mp.

77 tired*.mp.

78 fatigue*.mp.

79 cancer related fatigue.mp.

80 physical fatigue.mp.

81 mental fatigue.mp.

82 dump* syndrome.mp.

83 nutriti* impact symptom*.mp.

84 epigastric pain.mp.

85 epi gastric pain.mp.

86 hyp?glyc?emi*.mp.

87 pancrea* exocrine insufficien*.mp.

88 discomfort.mp.

89 reduced appetite.mp.

90 42 or 43 or 44 or 45 or 46 or 47 or 48 or 49 or 50 or 51 or 52 or 53 or 54 or 55 or 56 or 57 or 58 or 59 or 60 or 61 or 62 or 63 or 64 or 65 or 66 or 67 or 68 or 69 or 70 or 71 or 72 or 73 or 74 or 75 or 76 or 77 or 78 or 79 or 80 or 81 or 82 or 83 or 84 or 85 or 86 or 87 or 88 or 89

91 exp Malnutrition/

92 exp Thinness/

93 exp Body Composition/

94 exp Muscle, Skeletal/

95 exp Muscular Atrophy/

96 Nutritional Status/

97 sarcop?en*.mp.

98 body composition.mp.

99 musc* atrophy.mp.

100 musc* mass.mp.
101 musc* wast*.mp.
102 skeletal muscle.mp.
103 nutrition* status.mp.
104 malnutrition.mp.
105 protein energy malnutrition.mp.
106 under nutrition.mp.
107 under nourish*.mp.
108 cachexia.mp.
109 fat free mass.mp.
110 weight los*.mp.
111 lean mass*.mp.
112 malnourish*.mp.
113 undernourish*.mp.
114 cachectic.mp.
115 under weight.mp.
116 underweight.mp.
117 cancer cachexia.mp.

118	91 or 92 or 93 or 94 or 95 or 96 or 97 or 98 or 99 or 100 or 101 or 102 or 103 or 104 or 105 or 106 or 107 or 108 or 109 or 110 or 111 or 112 or 113 or 114 or 115 or 116 or 117
119	9 and 41 and 90 and 118
120	limit 119 to yr="2020 -Current"

SCOPUS

(TITLE-ABS-KEY ("oesophageal" OR "esophageal" OR "gastric" OR "stomach" OR "pancreatic" OR "pancreas ") AND TITLE-ABS-KEY ("cancer" OR "neoplasm*" OR "tumour*" OR "tumor*" OR "carcinoma*" OR "malignanc*") OR TITLE-ABS-KEY ("oesophageal adenocarcinoma*" OR "esophageal adenocarcinoma*" OR "gastric adenocarcinoma*" OR "pancreatic adenocarcinoma*") OR TITLE-ABS-KEY ("oesophageal dysplasia" OR "esophageal dysplasia" OR "gastric dysplasia" OR "pancreatic dysplasia") OR TITLE-ABS-KEY ("oesophageal lesion*" OR "esophageal lesion*" OR "gastric lesion*" OR "pancreatic lesion*")) AND (TITLE-ABS-KEY ("antineoplastic agen*" OR chemotherap* OR chemoradiotherap* OR "chemo radiotherap*" OR "chemo radio therap*" OR radiotherap* OR surger* OR operat* OR "radio* therap*" OR "chemo* therap*" OR "immun*therap*" OR "neo adjuvant*" OR "adjuvant treat*" OR "radiation therap*" OR "radiotherapeutic technique*" OR "radio therapeutic technique*" OR "anti neoplastic agent*" OR "anticancer drug*" OR "anti cancer drug*" OR "anticancer agent*" OR "anti cancer agent*" OR "antitumo?r drug*" OR "antitumo?r agent*" OR "anti tumo?r agent*" OR "tumo?r inhibitor" OR "anti tumo?r drug*")) AND (TITLE-ABS-KEY ("decreased appetite" OR "loss of appetite" OR "lack of appetite" OR anorexi* OR nause* OR vomit* OR emesis OR hyperemesis OR diarrh*ea OR ("loose" W/1 "motion*") OR constipat* OR mucositis OR "mucosa* inflamm*" OR "early satiety" OR dysgeusi* OR dysphagi* OR ("problem*" W/1 "swallow*") OR ("swallow*" W/1 "problem*") OR ("difficul*" W/1 "swallow*") OR ("dis*" W/1 "swallow*") OR "unsafe swallow*" OR "deglutition disorder" OR "dry mouth" OR symptom* OR "taste change*" OR pain OR bloat* OR reflux OR flatulence OR tired* OR fatigue* OR "cancer related fatigue" OR "physical fatigue" OR "mental fatigue" OR "dump* syndrome" OR "nutriti* impact symptom*" OR "epigastric pain" OR "epi gastric pain" OR (hypoglycemi* OR hyperglycemi* OR hypoglycaemi* OR hyperglycaemi*) OR "pancrea* exocrine insufficien*" OR discomfort OR "reduced appetite")) AND (TITLE-ABS-KEY (sarcop*en* OR "body composition" OR "musc* atrophy" OR "muscle mass" OR "musc* wast*" OR "skeletal muscle" OR "nutrition* status" OR malnutrition OR "protein energy malnutrition" OR "under nutrition" OR "under nourish*" OR cachexia OR "fat free mass" OR "weight los*" OR "lean mass*" OR malnourish* OR undernourish* OR cachectic OR "under weight" OR underweight OR "cancer cachexia")) AND PUBYEAR > 2019 AND PUBYEAR < 2026

Cochrane Library

#1	MeSH descriptor: [Esophageal Neoplasms] explode all trees

#2	MeSH descriptor: [Stomach Neoplasms] this term only
#3	MeSH descriptor: [Pancreatic Neoplasms] explode all trees
#4	(oesophag* OR esophag* OR gastr* OR stomach OR pancrea*) adj10 (cancer* OR tumo?r* OR neoplasm* OR carcinoma* OR malignan* OR adenocarcin*).af.
#5	#1 OR #2 OR #3 OR #4
#6	MeSH descriptor: [Radiotherapy] explode all trees
#7	MeSH descriptor: [Antineoplastic Agents] explode all trees
#8	MeSH descriptor: [General Surgery] this term only
#9	MeSH descriptor: [Drug Therapy] explode all trees
#10	antineoplastic NEXT agent*
#11	chemotherap*
#12	chemoradiotherap*
#13	chemo* NEXT radiotherap*
#14	chemo* NEXT radio* NEXT therap*
#15	radiotherap*
#16	surger*
#17	operat*
#18	radio* NEXT therap*
#19	chemo* NEXT therap*
#20	immun* NEXT therap*
#21	neo adjuvant
#22	adjuvant NEXT treat*
#23	radiation NEXT therap*
#24	radiotherapeutic NEXT technique*
#25	radio* NEXT therapeutic NEXT technique*
#26	anti NEXT neoplastic NEXT agent*
#27	anticancer NEXT drug*
#28	anti NEXT cancer NEXT drug*
#29	anticancer NEXT agent*
#30	anti* NEXT cancer NEXT agent*
#31	antitumo?r NEXT drug*
#32	antitumo?r NEXT agent*
#33	anti NEXT tumo?r NEXT agent*
#34	tumo?r NEXT inhibitor
#35	anti NEXT tumo?r NEXT drug*
#36	#6 OR #7 OR #8 OR #9 OR #10 OR #11 OR #12 OR #13 OR #14 OR #15 OR #16 OR #17 OR #18 OR #19 OR #20 OR #21 OR #22 OR #23 OR #24 OR #25 OR #26 OR #27 OR #28 OR #29 OR #30 OR #31 OR #32 OR #33 OR #34 OR #35
#37	MeSH descriptor: [Signs and Symptoms, Digestive] explode all trees
#38	MeSH descriptor: [Deglutition Disorders] explode all trees
#39	MeSH descriptor: [Mucositis] this term only
#40	MeSH descriptor: [Fatigue] explode all trees
#41	MeSH descriptor: [Pain] explode all trees
#42	"decreased appetite"
#43	"loss of appetite"
#44	"lack of appetite"
#45	"reduced appetite"

#46	anorexi*
#47	nause*
#48	vomit*
#49	emesis
#50	hyperemesis
#51	diarrh?ea
#52	loose NEXT motion*
#53	constipat*
#54	mucositis
#55	mucosa* NEXT inflamm*
#56	"early satiety"
#57	dysgeusi*
#58	dysphagi*
#59	(difficul* or problem*) NEAR/1 swallow*
#60	dis* NEAR/1 swallow*
#61	unsafe NEXT swallow*
#62	deglutition NEAR/1 disorder*
#63	dry mouth
#64	symptom*
#65	taste NEXT change*
#66	pain
#67	bloat*
#68	reflux
#69	flatulence
#70	tired*
#71	fatigue*
#72	dump* NEXT syndrome
#73	nutriti* NEXT impact NEXT symptom*
#74	(hypoglycemi* OR hyperglycemi* OR hypoglycaemi* OR hyperglycaemi*)
#75	pancrea* NEXT exocrine NEXT insufficien*
#76	discomfort
#77	#37 OR #38 OR #39 OR #40 OR #41 OR #42 OR #43 OR #44 OR #45 OR #46 OR #47 OR #48 OR #49 OR #50 OR #51 OR #52 OR #53 OR #54 OR #55 OR #56 OR #57 OR #58 OR #59 OR #60 OR #61 OR #62 OR #63 OR #64 OR #65 OR #66 OR #67 OR #68 OR #69 OR #70 OR #71 OR #72 OR #73 OR #74 OR #75 OR #76
#78	MeSH descriptor: [Body Composition] explode all trees
#79	MeSH descriptor: [Muscle, Skeletal] explode all trees
#80	MeSH descriptor: [Muscular Atrophy] explode all trees
#81	MeSH descriptor: [Nutritional Status] this term only
#82	MeSH descriptor: [Malnutrition] explode all trees
#83	MeSH descriptor: [Body Weight Changes] explode all trees
#84	MeSH descriptor: [Thinness] explode all trees
#85	sarcop?en*
#86	"body composition"
#87	muscl* NEXT atrophy
#88	"muscle mass"
#89	musc* NEXT wast*

#90	"skeletal muscle"
#91	nutrition* NEXT status
#92	malnutrition
#93	"protein energy malnutrition"
#94	under nutrition
#95	under NEXT nourish*
#96	cachexia
#97	"fat free mass"
#98	weight NEXT los*
#99	lean NEXT mass*
#100	malnourish*
#101	undernourish*
#102	cachectic
#103	"under weight"
#104	underweight
#105	#78 OR #79 OR #80 OR #81 OR #82 OR #83 OR #84 OR #85 OR #86 OR #87 OR #88 OR #89 OR #90 OR #91 OR #92 OR #93 OR #94 OR #95 OR #96 OR #97 OR #98 OR #99 OR #100 OR #101 OR #102 OR #103 OR #104
#106	#5 and #36 and #77 and #105 with Cochrane Library publication date Between Jan 2020 and Feb 2025
